# Effects of Adiponectin Including Reduction of Androstenedione Secretion and Ovarian Oxidative Stress Parameters *In Vivo*

**DOI:** 10.1371/journal.pone.0154453

**Published:** 2016-05-09

**Authors:** Fabio V. Comim, Karina Gutierrez, Alessandra Bridi, Guilherme Bochi, Raisa Chemeris, Melânia L. Rigo, Andressa Minussi P. Dau, Alfredo S. Cezar, Rafael Noal Moresco, Paulo Bayard Dias Gonçalves

**Affiliations:** 1 Department of Clinical Medicine, Federal University of Santa Maria (UFSM), Santa Maria, Brazil; 2 Laboratory of Biotechnology and Animal Reproduction (BioRep), Federal University of Santa Maria (UFSM), Santa Maria, Brazil; 3 Laboratory of Clinical Biochemistry, Federal University of Santa Maria (UFSM), Santa Maria, Brazil; Peiking University Third Hospital, CHINA

## Abstract

Adiponectin is the most abundantly produced human adipokine with anti-inflammatory, anti-oxidative, and insulin-sensitizing properties. Evidence from *in vitro* studies has indicated that adiponectin has a potential role in reproduction because it reduces the production of androstenedione in bovine theca cells *in vitro*. However, this effect on androgen production has not yet been observed *in vivo*. The current study evaluated the effect of adiponectin on androstenedione secretion and oxidative stress parameters in a rodent model. Seven-week-old female Balb/c mice (n = 33), previously treated with equine gonadotropin chorionic, were assigned to one of four different treatments: Group 1, control (phosphate-buffered saline); Group 2, adiponectin 0.1 μg/mL; Group 3, adiponectin 1.0 μg/mL; Group 4, adiponectin 5.0 μg/mL. After 24 h, all animals were euthanized and androstenedione levels were measured in the serum while oxidative stress markers were quantified in whole ovary tissue. Female mice treated with adiponectin exhibited a significant reduction (about 60%) in serum androstenedione levels in comparison to controls. Androstenedione levels decreased from 0.78 ± 0.4 ng/mL (mean ± SD) in controls to 0.28 ± 0.06 ng/mL after adiponectin (5 μg/mL) treatment (P = 0.01). This change in androgen secretion after 24 hours of treatment was associated with a significant reduction in the expression of CYP11A1 and STAR (but not CYP17A1). In addition, ovarian AOPP product levels, a direct product of protein oxidation, decreased significantly in adiponectin-treated mice (5 μg/mL); AOPP (mean ± SD) decreased to 4.3 ± 2.1 μmol/L in comparison with that of the controls (11.5 ± 1.7 μmol/L; P = 0.0003). Our results demonstrated for the first time that acute treatment with adiponectin reduced the levels of a direct oxidative stress marker in the ovary as well as decreased androstenedione serum levels *in vivo* after 24 h.

## Introduction

Adiponectin is the most abundantly secreted adipokine in the human body. This 30-Kd protein is recognized for its anti-diabetic, anti-inflammatory, and antiatherogenic properties [1–4]. Evidence in the literature has shown that adiponectin has beneficial cardiometabolic effects, such as increased fatty acid oxidation in muscle, augmented insulin sensitivity and reduction of reactive oxidative species (ROS)[1][5].

More recently, adiponectin has gained attention because of its potential role in reproductive physiology [6–12]. Studies *in vitro* have shown that steroid production in theca and granulosa layers are affected by this adipokine. In rat and bovine primary granulosa cell cultures co-treated with IGF-I, adiponectin augmented estradiol and progesterone secretion [13, 14]. Conversely, a decrease in the secretion of androgens (androstenedione) followed by a reduction in the expression of key steroidogenic enzymes such as CYP17A1 and CYP11A1 has been observed in bovine theca cell culture in response to adiponectin [7, 15]. The action of adiponectin is mainly mediated by its two receptors AdipoR1 and AdipoR2; suppression of gene expression by small interfering RNA (siRNA) for AdipoR1 and AdipoR2 can dramatically increase androgen secretion in bovine theca cells [7].

It remains unclear whether some of its *in vitro* inhibitory effects on the gonadal secretion of androgens could be dynamically observed in an *in vivo* model. Therefore, this study focused to address two simple aims: 1) Can acute adiponectin administration reduce ovarian androstenedione levels in a rodent model? 2) What is the effect of this treatment on oxidative stress markers in the ovary? This last question was based on the hypothesis that adiponectin can decrease ROS directly in the gonad. Moreover, previous reports have pointed the negative impact of dysregulation of oxidative stress in the functioning of theca cells and ovulation in rodents[16, 17].

As shown below, intraperitoneal administration of adiponectin (0.1 μg/mL, 1.0 μg/mL, or 5.0 μg/mL) significantly reduced androstenedione secretion and levels of direct oxidative stress marker, AOPP, in Balb C female mice. To the best of our knowledge, this is the first study to confirm the findings of previous *in vitro* studies that had demonstrated the activity of adiponectin to regulate ovarian androgen secretion.

## Materials and Methods

### Animals

Balb/C adult (seven weeks old) female mice were used in this study. They were housed in polypropylene cages with water and food ad libitum in an animal facility equipped with a 12:12 h light-dark cycle and under a controlled temperature (22 ± 2°C). Animals were kept in an enriched environment to enhance living conditions in agreement with the National Guidelines of National Council of Control of Animal Experimentation (CONCEA, Brazil). All procedures were carried out with the approval of the Committee on Ethics in the Use of Animals from the Federal University of Santa Maria (CEUA-UFSM) number 090-2012-2013.

### Experimental protocol

Overall, 33 female mice received equine gonadotropin chorionic (eCG) (Folligon; Intervet Schering) 10 UI intra-peritoneal (IP) 2 days before the following treatments (200 μL intra-peritoneal): 1) Group 1 (n = 9), control (phosphate-buffered saline); 2) Group 2 (n = 9), human adiponectin 0.1 μg/mL; 3) Group 3 (n = 8), human adiponectin 1.0 μg/mL; 4) Group 4 (n = 7), human adiponectin 5.0 μg/mL. The total blood volume of each mouse was calculated using the formula [58.5 mL/kg x weight (kg)]. After 24 h, all animals were euthanized, and their blood and ovary tissue were collected. Arbitrary doses of adiponectin in a range of 50 times (from 0.1 μg/mL to 5 μg/mL) were defined for a challenge in mice, using as a reference studies previously published for other purposes [18–21]. The use of equine chorionic gonadotropin (eCG) was performed to promote periovulatory maturation in mice, given the fact that studies *in vitro* had used mainly large antral follicles in the periovulatory period [7, 15, 17]. In addition, it may helped to avoid a possible influence of different estrous cycles in ovarian oxidative stress or androgen secretion.

### Adiponectin treatment and oxidative stress markers

Human recombinant adiponectin was from Sigma-Aldrich, USA (SRP4901) and administrated intraperitoneally. Nitrogen oxide (NOx) levels, ferric reducing ability of plasma (FRAP), and the products of advanced protein oxidation (AOPP) were evaluated in whole homogenized ovaries using the Cobas Mira^®^ automated analyzer (Roche Diagnostics, Basel, Switzerland) as previously described [22–24].

### ELISA

Androstenedione levels were measured in serum using a specific ELISA for mouse models (ABIN627568, Antibodies On-line, USA). The intra-assay and inter-assay coefficient of variation were 10 and 9.5%, respectively.

### Real Time—PCR

Isolation of mRNA was prepared from the whole ovary tissue extracts using Trizol^®^ (Thermo Fischer, Life Technologies do Brasil Ltda, Brazil) according to the manufacturer´s instructions. Complementary DNA (cDNA) was synthesized from 500 ng RNA, initially treated with 0.1 U DNase, Amplification Grade (Life Technologies, Burlington, Canada) for 5 min at 37°C. Subsequently, samples were submitted to DNase inactivation at 65°C for 10 min and incubated in a final volume of 20 μl with iScript cDNA Synthesis Kit (BioRad, Hercules, CA, USA). CDNA synthesis was then performed in three steps: 25°C– 5 min, 42°C– 30 min and 85°C– 5 min[25]. Primers used for qPCR and RT-PCR reactions were designed with the software Primer 3, version 4.0 (http://frodo.wi.mit.edu) (Whitehead Institute for Biomedical Research, Massachusetts, USA) using the database from NCBI library (http://www.ncbi.nlm.nih.gov/nucleotide). The following primers (an accession numbers) were used for RT-PCR, as follows: 1) CYP17A1 (NM_007809): forward 5′-TCAAGGTGACAATCAGAAACGC-3’ and reverse 3’-AAGAAATAGGCCAGGATGAGCA-5’; 2) CYP11A1 (NM_019779): forward 5’- GTCTACCAGATGTTCCACACCA-3’ and reverse: 3’- CCAGGAGGCTATAAAGGACACC-5’; 3) STAR (NM_011485): forward 5’- TGCCGAAGACAATCATCAACCA-3’ and reverse 3’- GCTTCCTGTGAGAGCTTCCAAT-5’; 4) RPL4 (NM_024212): forward 5’-CAGAGAATGAGAGCTGGCAAGG-3’ and reverse 3’-TGCCATACAGCTCATCCAACTT-5’. The quantitative polymerase chain reactions (qPCR) were executed in a CFX384 thermocycler (BioRad) using GoTaq^®^ qPCR Master Mix (Promega, Promega Corporation, Madison, USA). The protocol for Two-step qPCR was included an initial denaturation at 95°C for 5 min succeeded by 40 cycles of denaturation at 95°C for 15 sec and annealing/extension at 60°C for 30 sec[25]. Amplification efficiency of the RT-PCR reactions was between 90 and 110%. Comparison of gene expression was performed by ΔΔCq method and having RPL4 as a reference gene.

### Statistical analysis

Data were described as mean ± SEM or median (IQR 25, 75). Differences between the groups were distinguished using the Student’s t-test (normal distribution) or Mann-Whitney test (if asymmetrical distribution). A statistically significant association was defined when P<0.05. The analysis and graphs were performed using the statistical program GraphPad Prism 6.0 (San Diego CA, USA).

## Results

### Effect on androstenedione secretion (24 h)

**Fig 1:**
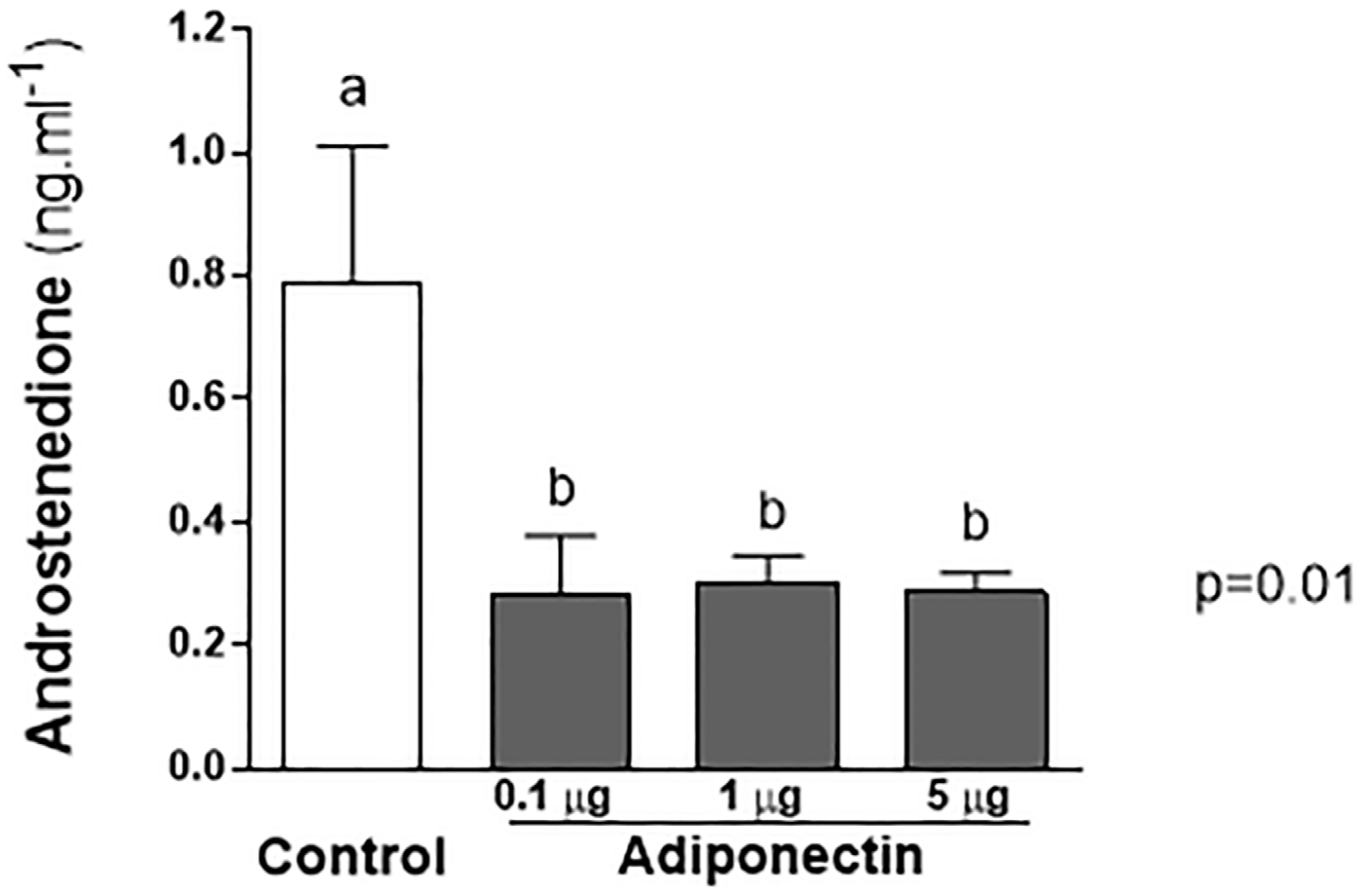
Effect of adiponectin on reduction of androstenedione serum levels (24h). Mice previously synchronized with equine gonadotropin chorionic (eCG), were submitted to one of the four different treatments: 1) Group 1- control (PBS), Group 2—adiponectin 0.1 μg/mL, Group 3—adiponectin 1 μg/mL, and Group 4—adiponectin 5 μg/mL. After 24 h the animals were euthanized and serum levels of androstenedione evaluated (mean ± SEM). There was a statistically significant reduction in adiponectin treated groups (ANOVA p = 0.01).

Androgen serum levels were markedly decreased (about 60%) after 24-h treatment with adiponectin, as shown in Fig 1. The levels of androstenedione (mean ± SEM) of the control group were 0.79 ± 0.22 ng/mL, and that of adiponectin groups at 0.1, 1.0, and 5.0 μg/mL were, respectively, 0.28 ± 0.09, 0.30 ± 0.04, and 0.28 ± 0.03 ngmL, (P = 0.01).

### Effect on ovarian key steroidogenic enzymes and StAR

**Fig 2:**
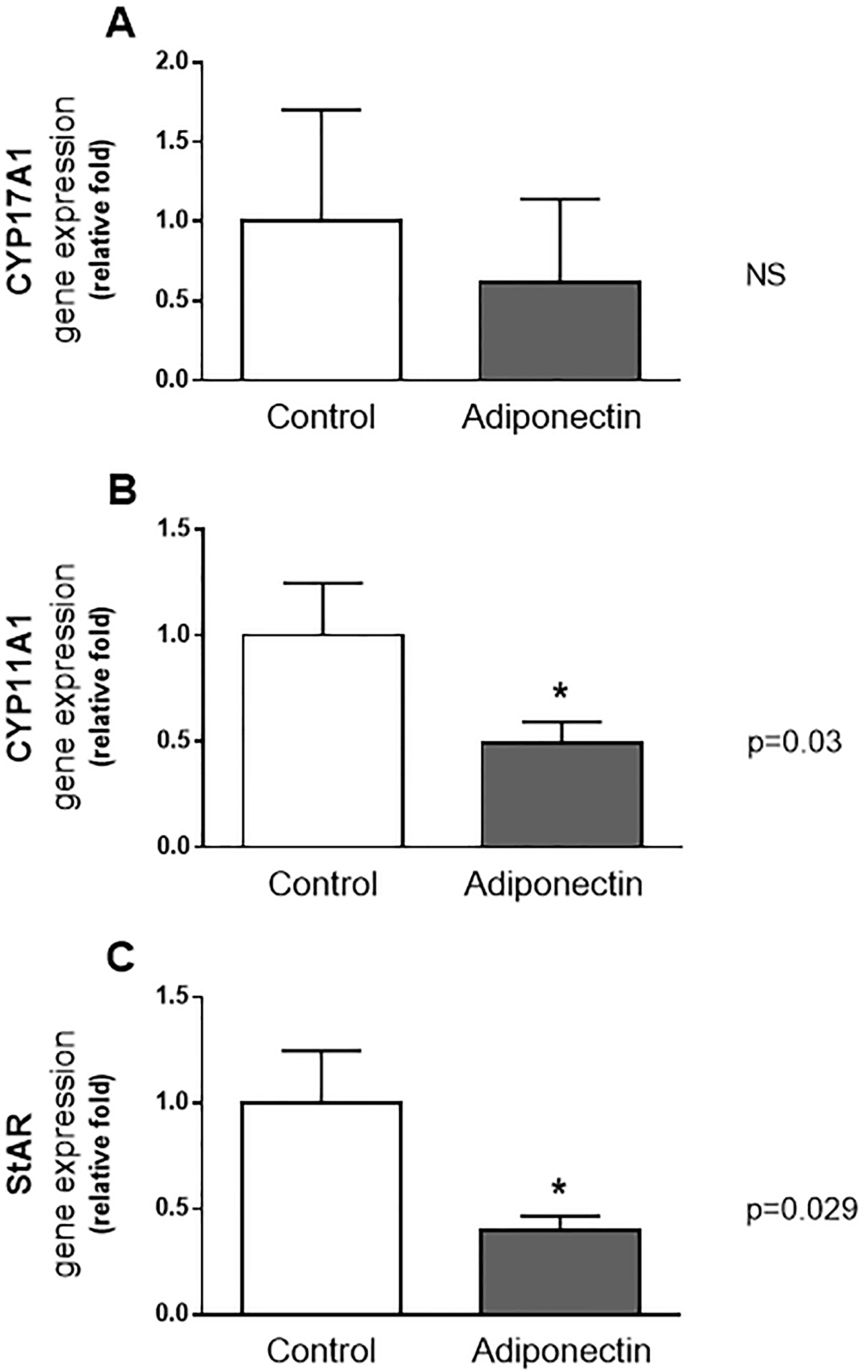
Gene expression of CYP17A1, CYP11A1 and STAR in ovaries after treatment of adiponectin (24h). In the animals treated with 1 μg/mL of adiponectin, a reduction in gene expression of CYP11A1(p = 0.03) (A), STAR (p = 0.029) (B), but not CYP17A1(C) was observed. Results were normalized (control = 1,0 arbitrary units).

Fig 2 shows the effects of treatment with adiponectin (at 1 μg/mL) through the reduction in the gene expression of steroidogenic enzymes and protein in the full ovary in comparison to the control group. It was observed a significant decrease in CYP11A1 in adiponectin group, with mean ± SEM of gene expression (in arbitrary units) of 0.51 ± 0.1 versus 1.52± 0.2 in the control group (p = 0.03) (Fig 2B). A similar reduction was seen with STAR, where the mean ± SEM of gene expression (in arbitrary units) was 0.45 ± 0.07 in adiponectin group and 1.14 ± 0.28 (p = 0.029) in the controls (Fig 2C). Nevertheless, no differences in the control group were identified concerning CYP17A1; the mean ± SEM of expression (in arbitrary units) for this gene was 0.1548 ± 0.10 in control group against 0.09 ± 0.07 in adiponectin group (p = 0.7) (Fig 2A).

### Effect on oxidative stress (24 h)

**Fig 3:**
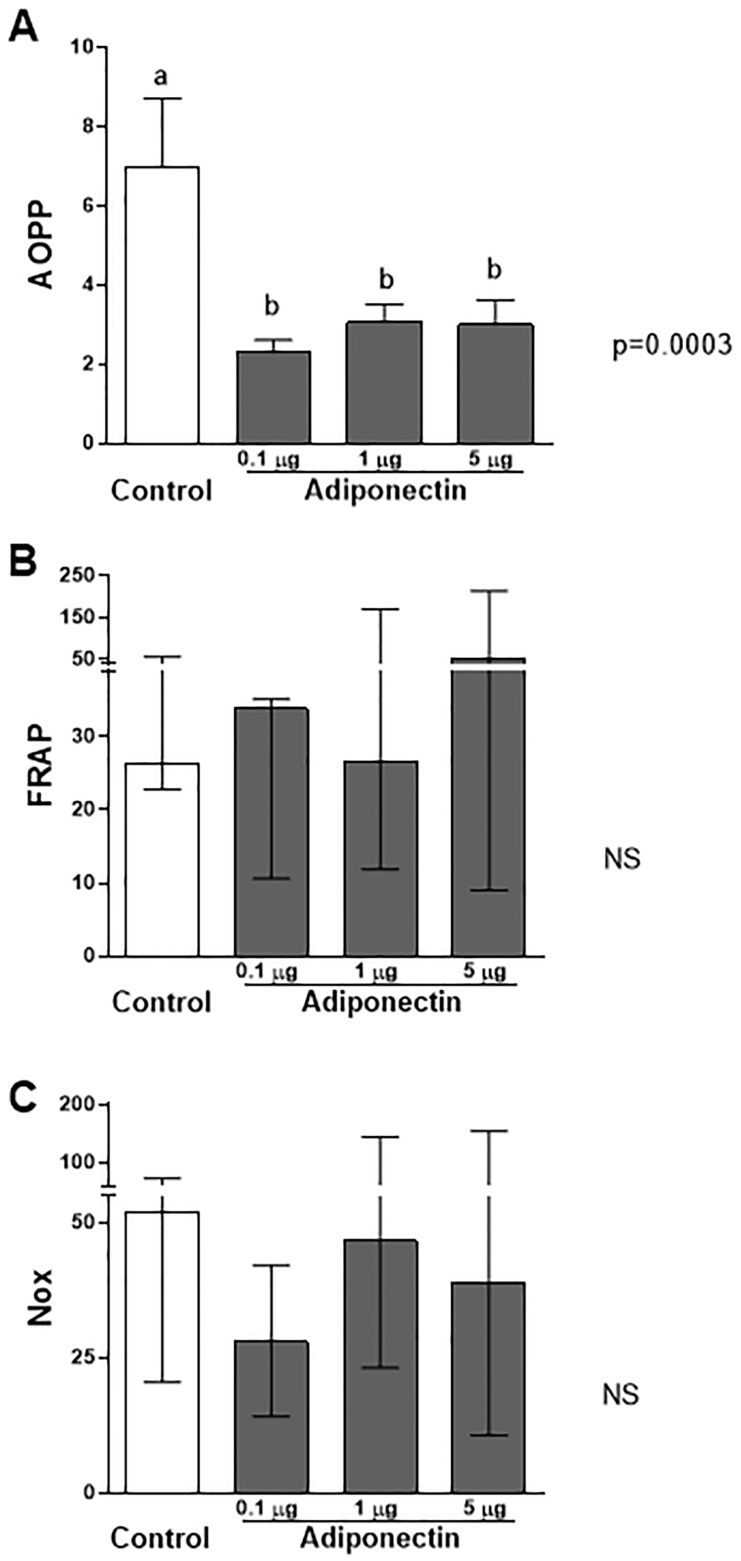
Effects of the different doses of adiponectin on markers of direct oxidative stress (AOPP)(A) and anti-oxidant capacity (FRAP and Nox)(B and C). (A)Compared to control cells, adiponectin treatment (in all doses tested) decreased the AOPP levels (ANOVA, P = 0.0003). (B and C) No differences were seen in terms of anti-oxidant capacity markers, namely FRAP and NoX. Data shown represents mean ± SEM (A) or median (IQ 25–75%) (B and C).

Oxidative stress markers were employed to analyze the potential benefits of adiponectin against reactive oxygen species (ROS) in the ovary. We estimated the direct damage from oxidative stress through the analysis of products of advanced protein oxidation (AOPP). The antioxidant capacity was defined by measuring NOx levels and FRAP. Adiponectin decreased the ovarian AOPP levels, indicating reduced oxidative stress (P = 0.0003; Fig 3A). The AOPP level of the control group was 6.97 ± 1.7 (mean ± SEM), and that of the adiponectin groups at 0.1, 1.0, and 5.0 μg/mL was 2.29 ± 0.31, 3.06 ± 0.44, and 3.0 ± 0.62, respectively, (P = 0.0003).

In relation to antioxidant capacity (FRAP and Nox), the differences observed were not statistically significant (Fig 3B and 3C). The median FRAP values (IQ 25–75%) were 26.2 (22.76–56.74), 33.8 (10.6–35.2), 26.6 (11.88–169.5), and 52.29 (9–213.5) in the control group, adiponectin group 0.1 μg/mL, adiponectin group 1.0 μg/mL, and adiponectin group 5.0 μg/mL (P = 0.45). The median Nox values for the control group (IQ 25–75%) corresponded to 51.8 (20.5–73.5), while that for the adiponectin groups (0.1, 1.0, and 5.0 μg/mL) corresponded to 28.0 (14.2–42.2), 46.73 (23.2–144.8), and 38.8 (10.7–154.6), respectively (P = 0.29; Fig 3C).

## Discussion

Adiponectin and its receptors have been identified in reproductive tissues of different species including human, porcine and rodents[6, 13, 26–29]. To date, some reports have documented a potential role of adiponectin in ovulation, fertility and embryo development, although mechanisms remain in part elusive [30, 31].

The present study, for the first time, provided evidence that the administration of recombinant human adiponectin remarkably decreased ovarian androstenedione levels *in vivo*, using Balb/C female mice. This change in androgen secretion after 24 hours of treatment was associated with a significant reduction in the expression of CYP11A1 and STAR (but not CYP17A1). CYP11A1 is recognized as the rate-limiting enzyme in steroidogenesis, while CYP17A1 represents the rate-limiting enzyme to sex steroids [32].

Our results agreed with previous studies *in vitro* that showed a reduction of androstenedione secretion in bovine theca cells in the presence of LH and insulin after 24 hours [7, 15]. However, it claimed the attention the lack of dose-response in our study, given the marked effect of lower doses (e.g. 0.1 μg/mL) in comparison to studies *in vitro*, which treatments usually reached 3 μg/mL. In respect to this situation, two aspects should be considered. The first one is the probable co-action of adiponectin at extra-ovarian sites, such as hypothalamus and pituitary, promoting a stronger decline in androgens. Indeed, previous studies *in vitro* have demonstrated that adiponectin can also influence the hypothalamic-pituitary-gonadal axis [33]. Experiments using GTI-7 hypothalamic cells have shown that adiponectin may lower gonadotropin releasing hormone (GnRH) activity as a consequence of AMPK activation followed by downregulation of extracellular signal-regulated kinase (ERK) pathway [34, 35]. In a rat pituitary primary cell culture, adiponectin caused a 50% decrease in luteinizing hormone (LH) secretion [36]. Therefore, we speculate that other systems such as the hypothalamus and the pituitary glands were affected, resulting in reduced GnRH and gonadotropin levels and possibly contributing to the reduction of androgen secretion. The second aspect reports to the study of *Caminos et al*., which observed with minimal doses of adiponectin (such as 0.01 μg/mL per gram of incubated tissue) a dramatic decrease in testosterone production after 3 hours in incubated rat testis *ex vivo* [20]. This suggests a possible interaction of other cellular structural components of the ovary/testis, may influence or favor adiponectin action.

Another important subject of this study was the impact of adiponectin on oxidative stress in the ovary. As shown, adiponectin administration caused a reduction (in extracts of the whole ovary) of products of advanced protein oxidation (AOPP), a direct marker of action of ROS. Recent evidence from the transcriptome profiling of bovine theca interna from large follicles by Hatzirodos *et al*. (2014) suggests that some degree of oxidative stress is normal and may be due to steroidogenesis or even the activity of NADPH oxidases in the vascular endothelium [37]. Nevertheless, it is not known currently whether a “basal” production of ROS is required for normal functioning of an ovary. Some studies have connected a higher oxidative stress and lower antioxidant capacity with infertility. In swine, for example, experimental hypoxia leading to follicular atresia was associated with changes in ROS[38, 39]. Studies in mice have shown that excessive concentrations of ROS were deleterious to oocyte development culminating with degeneration and cell death of zygote[40–42].

Our study presented several limitations. Firstly, it did not address the impact of adiponectin in different moments of the estral cycle in mice. Secondly, it analyzed just the levels of a single androgen (which may be converted into estrogen). Thirdly, despite the impact on steroid production focused on the ovary, this study not explored possible action at other sites as the pituitary or adrenal. On the other hand, some strengths of the present report include a broad range of adiponectin concentrations tested (0.1–5.0 μg/mL) that surpasses the physiological limits [43]. Serum androstenedione was obtained by a specific murine ELISA assay.

The results presented also show some implications for polycystic ovary syndrome (PCOS), the most frequent cause of androgen excess in women at menacme [44, 45]. PCOS is characterized by chronic inflammation, increased oxidative stress parameters, and reduced levels of adiponectin [6, 9, 10, 31, 44, 46]. In addition to lower levels of adiponectin, there is a decrease in ADIPOR1 and ADIPOR2 receptors in the theca of polycystic ovaries compared to normal ovaries [7]. The evidence that adiponectin can lower androgen levels (and vice-versa) reinforces a possible role for this adipokine as a mediator between metabolic and reproductive features in humans as suggested by previous reports [7, 15, 47]. The present study opens a window for other important experiments including the impact of adiponectin in androgenized animals (a model of PCOS) as well in the presence of obesity.

In conclusion, our results provide initial evidence of the ability of adiponectin to reduce ovarian androgen levels *in vivo*, corroborating previous reports employing bovine theca cell culture *in vitro*. In addition, the present study suggests a novel role for adiponectin to reduce oxidative stress (AOPP) in the ovarian tissue. Further studies are necessary to confirm how these factors operate in the ovary and hypothalamic-pituitary compartments.
